# Supplementary figures and images for: Decoupling AMPK from fatty acid synthesis allows maintenance of fitness late in life
Source: eLife. 2026 Jul 31;15:RP111611. doi: 10.7554/eLife.111611 (PMC13427345; doi:10.7554/eLife.111611)

2 biological replicate experiments were run

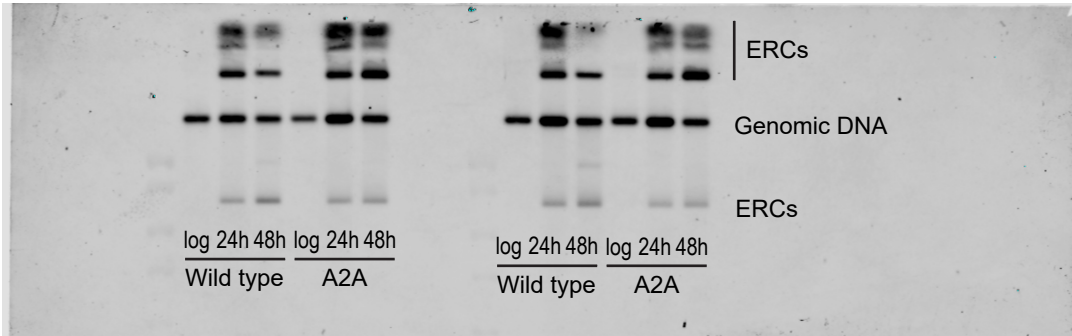

Ladder

Supplement: Figure 2—figure supplement 1—source data 1. [file elife-111611-fig2-figsupp1-data1.zip › Figure 3-figure supplement 1-source data 2.pdf]

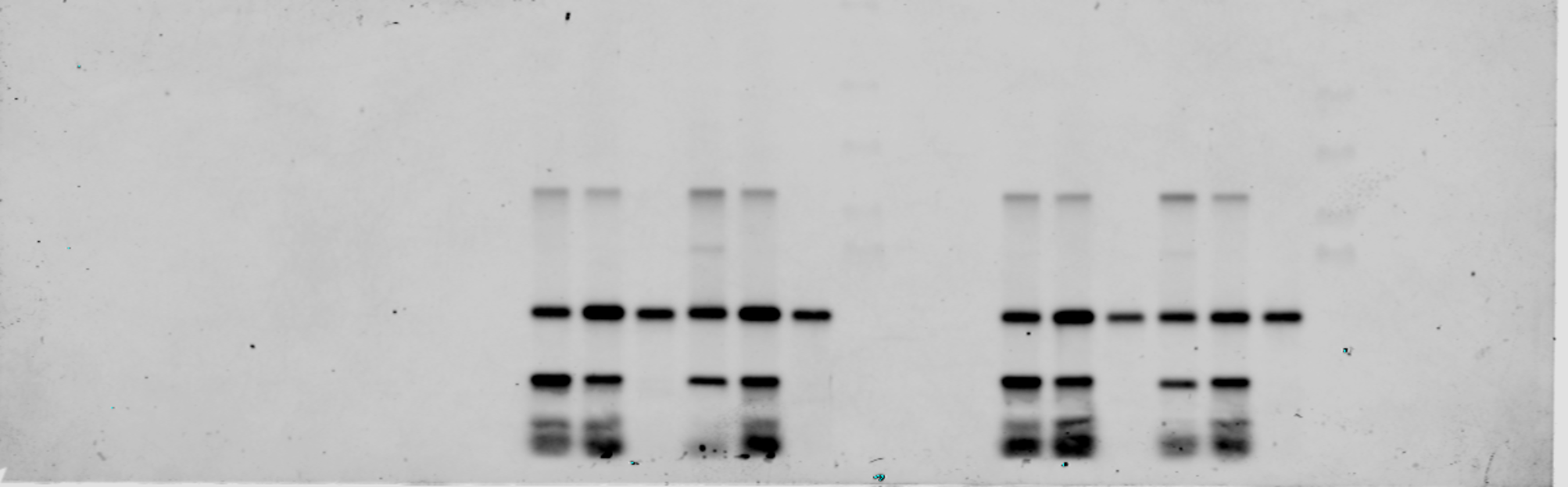

Supplement: Figure 2—figure supplement 1—source data 2. [file elife-111611-fig2-figsupp1-data2.zip › Hanane Megan blot - NTS2 Bap probe5.tif]
