# Supplementary material for: Decoupling AMPK from fatty acid synthesis allows maintenance of fitness late in life
Source: eLife. 2026 Jul 31;15:RP111611. doi: 10.7554/eLife.111611 (PMC13427345; doi:10.7554/eLife.111611)
Supplement: Supplementary file 1. [file elife-111611-supp1.docx]

**Supplementary file 1: Strains used in this work.**

All strains are diploid derivatives of the MEP system (47). TOM70-GFP and RPL13a-mCherry markers are heterozygous to avoid growth defect. Individual genes in genotypes are homozygous unless indicated otherwise with a “/”

| DH18 | WT-RPL13a  MEP Tom70-GFP Rpl13a-mCherry | *ade2::hisG his3 leu2 met15D::ADE2/+ lys2/+ ura3D0 trp1D63 hoD::SCW11pr-Cre-EBD78-NatMX loxP-UBC9-loxP-LEU2 loxP-CDC20-Intron-loxP-HPHMX Tom70-GFP-TRP1/+ RPL13A-mCherry-Kan/+* |
| --- | --- | --- |
| JH1551 | **ACC1^S1157A^-RPL13a**  MEP Tom70-GFP Rpl13a-mCherry acc1-S1157A het. | *ade2::hisG his3 leu2 met15D::ADE2/+ lys2/+ ura3D0 trp1D63 hoD::SCW11pr-Cre-EBD78-NatMX loxP-UBC9-loxP-LEU2 loxP-CDC20-Intron-loxP-HPHMX Tom70-GFP-TRP1/+ RPL13A-mCherry-Kan/+ acc1-S1157A/+* |
| DH83 | **P_GPD_-SAK1-RPL13a**  MEP diploid TOM70-GFP RPL13A-mCherry SAK1 oe | *ade2::hisG his3 leu2 met15D::ADE2/+ lys2/+ ura3D0 trp1D63 hoD::SCW11pr-Cre-EBD78-NatMX loxP-UBC9-loxP-LEU2 loxP-CDC20-Intron-loxP-HPHMX Tom70-GFP-TRP1/+ RPL13A-mCherry-Kan/+ KanMX6-Pgdp-SAK1/+* |
| JH1552 | **A2A-RPL13a**  MEP Tom70-GFP Rpl13a-mCherry SAK1 oe acc1-S1157A het. | *ade2::hisG his3 leu2 met15D::ADE2/+ lys2/+ ura3D0 trp1D63 hoD::SCW11pr-Cre-EBD78-NatMX loxP-UBC9-loxP-LEU2 loxP-CDC20-Intron-loxP-HPHMX Tom70-GFP-TRP1/+ RPL13A-mCherry-Kan/+ KanMX6-Pgdp-SAK1/+ acc1-S1157A/+* |
| DH192 | **COX9Δ**  MEP diploid TOM70-GFP RPL13A-mCherry cox9Δ | *ade2::hisG his3 leu2 met15D::ADE2/+ lys2/+ ura3D0 trp1D63 hoD::SCW11pr-Cre-EBD78-NatMX loxP-UBC9-loxP-LEU2 loxP-CDC20-Intron-loxP-HPHMX Tom70-GFP-TRP1/+ RPL13A-mCherry-Kan/+ cox9::URA3* |
| MU19 | **COX9Δ P_GPD_-SAK1**  MEP diploid COX9Δ SAK1oe (TOM70-GFP RPL13A-mCherry) | *ade2::hisG his3 leu2 met15D::ADE2/+ lys2/+ ura3D0 trp1D63 hoD::SCW11pr-Cre-EBD78-NatMX loxP-UBC9-loxP-LEU2 loxP-CDC20-Intron-loxP-HPHMX Tom70-GFP-TRP1/+ RPL13A-mCherry-Kan/+ cox9::URA3 KanMX6-Pgdp-SAK1/+* |
| JH1676 | **COX9Δ A2A**  cox9Δ/cox9Δ SAK1 oe acc1-S1157A Tom70-GFP Rpl13-mCherry | *ade2::hisG his3 leu2 lys2/+ met15::ADE2/+ ura3D0 trp1D63 hoD::SCW11pr-Cre-EBD78-NatMX loxP-UBC9-loxP-LEU2 loxP-CDC20-Intron-loxP-HPHMX KanMX6-Pgdp-SAK1/+ acc1-S1157A/+ cox9::URA3/cox9::URA3 TOM70-GFP-Kan/+ RPL13a-mCherrry-TRP1/+* |
| MU38 | **MPC1Δ**  MPC1Δ MEP diploid (TOM70-GFP VPH1-mCherry) | *ade2::hisG his3 leu2 met15D::ADE2/+ lys2/+ ura3D0 trp1D63 hoD::SCW11pr-Cre-EBD78-NatMX loxP-UBC9-loxP-LEU2 loxP-CDC20-Intron-loxP-HPHMX Tom70-GFP-TRP1/+ VPH1-mCherry-Kan/+ MPC1::HIS* |
| MU39 | **MPC1Δ P_GPD_-SAK1**  MPC1Δ SAK1 oe MEP diploid (TOM70-GFP VPH1-mCherry) | *ade2::hisG his3 leu2 met15D::ADE2/+ lys2/+ ura3D0 trp1D63 hoD::SCW11pr-Cre-EBD78-NatMX loxP-UBC9-loxP-LEU2 loxP-CDC20-Intron-loxP-HPHMX Tom70-GFP-TRP1/+ VPH1-mCherry-Kan/+ KanMX6-Pgdp-SAK1/+ MPC1::HIS* |
| MU40 | **MPC1Δ A2A**  MPC1Δ + A2A MEP diploid (TOM70-GFP VPH1-mCherry) | *ade2::hisG his3 leu2 met15D::ADE2/+ lys2/+ ura3D0 trp1D63 hoD::SCW11pr-Cre-EBD78-NatMX loxP-UBC9-loxP-LEU2 loxP-CDC20-Intron-loxP-HPHMX Tom70-GFP-TRP1/+ VPH1-mCherry-Kan/+ KanMX6-Pgdp-SAK1/+ acc1-S1157A/+ MPC1::HIS* |
| HH40HH40 | **MLS1Δ**  MLS1 Δ MEP diploid (Tom70-GFP RPL13A-mCherry) | *ade2::hisG his3 leu2 met15D::ADE2/+ lys2/+ ura3D0 trp1D63 hoD::SCW11pr-Cre-EBD78-NatMX loxP-UBC9-loxP-LEU2 loxP-CDC20-Intron-loxP-HPHMX Tom70-GFP-TRP1/+ RPL13A-mCherry-Kan/+ MLS1::URA3* |
| MU31 | **MLS1Δ P_GPD_-SAK1**  MLS1Δ SAK1 oe MEP diploid (Tom70-GFP RPL13A-mCherry) | *ade2::hisG his3 leu2 met15D::ADE2/+ lys2/+ ura3D0 trp1D63 hoD::SCW11pr-Cre-EBD78-NatMX loxP-UBC9-loxP-LEU2 loxP-CDC20-Intron-loxP-HPHMX Tom70-GFP-TRP1/+ RPL13A-mCherry-Kan/+ KanMX6-Pgdp-SAK1/+ MLS1::URA3* |
| MU59 | **MLS1Δ A2A**  MLS1 Δ + Sak1 oe + ACC1 S1157A MEP diploid (Tom70-GFP RPL13A-mCherry) | *ade2::hisG his3 leu2 met15D::ADE2/+ lys2/+ ura3D0 trp1D63 hoD::SCW11pr-Cre-EBD78-NatMX loxP-UBC9-loxP-LEU2 loxP-CDC20-Intron-loxP-HPHMX Tom70-GFP-TRP1/+ RPL13A-mCherry-Kan/+ KanMX6-Pgdp-SAK1/+ acc1-S1157A/+ MLS1::URA3* |
| HH47 | **SFC1Δ**  SFC1Δ MEP diploid (TOM70-GFP + Rpl13a-mCherry) | *ade2::hisG his3 leu2 met15D::ADE2/+ lys2/+ ura3D0 trp1D63 hoD::SCW11pr-Cre-EBD78-NatMX loxP-UBC9-loxP-LEU2 loxP-CDC20-Intron-loxP-HPHMX Tom70-GFP-TRP1/+ RPL13A-mCherry-Kan/+ SFC1::URA3* |
| MU32 | **SFC1Δ P_GPD_-SAK1**  SFC1Δ SAK1 oe MEP diploid (Tom70-GFP RPL13A-mCherry) | *ade2::hisG his3 leu2 met15D::ADE2/+ lys2/+ ura3D0 trp1D63 hoD::SCW11pr-Cre-EBD78-NatMX loxP-UBC9-loxP-LEU2 loxP-CDC20-Intron-loxP-HPHMX Tom70-GFP-TRP1/+ RPL13A-mCherry-Kan/+ KanMX6-Pgdp-SAK1/+ SFC1::URA3* |
| MU66 | **CAT2Δ MLS1Δ**  CAT2Δ + MLS1 Δ MEP diploid (Tom70-GFP RPL13A-mCherry) | *ade2::hisG his3 leu2 met15D::ADE2/+ lys2/+ ura3D0 trp1D63 hoD::SCW11pr-Cre-EBD78-NatMX loxP-UBC9-loxP-LEU2 loxP-CDC20-Intron-loxP-HPHMX Tom70-GFP-TRP1/+ RPL13A-mCherry-Kan/+ MLS1::URA3 CAT2::HIS* |
| MU45 | **CAT2Δ MLS1Δ P_GPD_-SAK1**  CAT2Δ + MLS1Δ + SAK1o/e MEP diploid (Tom70-GFP RPL13A-mCherry) | *ade2::hisG his3 leu2 met15D::ADE2/+ lys2/+ ura3D0 trp1D63 hoD::SCW11pr-Cre-EBD78-NatMX loxP-UBC9-loxP-LEU2 loxP-CDC20-Intron-loxP-HPHMX Tom70-GFP-TRP1/+ RPL13A-mCherry-Kan/+ KanMX6-Pgdp-SAK1/+ MLS1::URA3 CAT2::HIS* |
| MU69 | **CAT2Δ MLS1Δ A2A**  CAT2Δ MLS1Δ SAK1 oe acc1-S1157A MEP diploid (Tom70-GFP RPL13A-mCherry) | *ade2::hisG his3 leu2 met15D::ADE2/+ lys2/+ ura3D0 trp1D63 hoD::SCW11pr-Cre-EBD78-NatMX loxP-UBC9-loxP-LEU2 loxP-CDC20-Intron-loxP-HPHMX Tom70-GFP-TRP1/+ RPL13A-mCherry-Kan/+ KanMX6-Pgdp-SAK1/+ acc1-S1157A/+ MLS1::URA3 CAT2::HIS* |
| HH88 | **SIP2Δ**  Sip2Δ MEP diploid TOM70-GFP VPH1-mCherry | *ade2::hisG his3 leu2 met15D::ADE2/+ lys2/+ ura3D0 trp1D63 hoD::SCW11pr-Cre-EBD78-NatMX loxP-UBC9-loxP-LEU2 loxP-CDC20-Intron-loxP-HPHMX Tom70-GFP-TRP1/+ VPH1-mCherry-Kan/+ Sip2::URA3* |
| HH108 | **SIP2Δ P_GPD_-SAK1**  Sip2Δ MEP diploid SAK1 oe (Tom70-GFP + VPH1-mCherry) | *ade2::hisG his3 leu2 met15D::ADE2/+ lys2/+ ura3D0 trp1D63 hoD::SCW11pr-Cre-EBD78-NatMX loxP-UBC9-loxP-LEU2 loxP-CDC20-Intron-loxP-HPHMX Tom70-GFP-TRP1/+ VPH1-mCherry-Kan/+ KanMX6-Pgdp-SAK1/+ Sip2::URA3* |
| HH87 | **SIP2Δ A2A**  Sip2Δ SAK1 oe acc1-S1157A (Tom70-GFP + VPH1-mCherry) | *ade2::hisG his3 leu2 met15D::ADE2/+ lys2/+ ura3D0 trp1D63 hoD::SCW11pr-Cre-EBD78-NatMX loxP-UBC9-loxP-LEU2 loxP-CDC20-Intron-loxP-HPHMX Tom70-GFP-TRP1/+ VPH1-mCherry-Kan/+ KanMX6-Pgdp-SAK1/+ acc1-S1157A/+ Sip2::URA3* |
| MU64 | **CAT2Δ**  MEP diploid (Tom70-GFP RPL13A-mCherry) | *ade2::hisG his3 leu2 met15D::ADE2/+ lys2/+ ura3D0 trp1D63 hoD::SCW11pr-Cre-EBD78-NatMX loxP-UBC9-loxP-LEU2 loxP-CDC20-Intron-loxP-HPHMX Tom70-GFP-TRP1/+ RPL13A-mCherry-Kan/+ CAT2::HIS* |
| MU49 | **SIP2Δ P_GPD_-SAK1 ALD6Δ**  ALD6Δ + Sip2Δ + SAK1 oe MEP diploid (TOM70-GFP VPH1-mCherry) | *ade2::hisG his3 leu2 met15D::ADE2/+ lys2/+ ura3D0 trp1D63 hoD::SCW11pr-Cre-EBD78-NatMX loxP-UBC9-loxP-LEU2 loxP-CDC20-Intron-loxP-HPHMX Tom70-GFP-TRP1/+ VPH1-mCherry-Kan/+ KanMX6-Pgdp-SAK1 Sip2::URA3 ALD6::HIS* |
| HH139 | **SIP2^3R^**  MEP DIPLOID TOM70-GFP VPH1-mCherry | *ade2::hisG his3 leu2 met15D::ADE2/+ lys2/+ ura3D0 trp1D63 hoD::SCW11pr-Cre-EBD78-NatMX loxP-UBC9-loxP-LEU2 loxP-CDC20-Intron-loxP-HPHMX Tom70-GFP-TRP1/+ VPH1-mCherry-Kan/+ sip2-K12R,K16R,K17R* |
| HH140 | **SIP2^3R^ P_GPD_-SAK1**  MEP DIPLOID | *ade2::hisG his3 leu2 met15D::ADE2/+ lys2/+ ura3D0 trp1D63 hoD::SCW11pr-Cre-EBD78-NatMX loxP-UBC9-loxP-LEU2 loxP-CDC20-Intron-loxP-HPHMX Tom70-GFP-TRP1/+ VPH1-mCherry-Kan/+ sip2-K12R,K16R,K17R KanMX6-Pgdp-SAK1/+* |
| HH141 | **SIP2^3R^ A2A**  MEP  P_GPD_-SAK1 acc1^S1157A^  (TOM70-GFP VPH1-mCherry) | *ade2::hisG his3 leu2 met15D::ADE2/+ lys2/+ ura3D0 trp1D63 hoD::SCW11pr-Cre-EBD78-NatMX loxP-UBC9-loxP-LEU2 loxP-CDC20-Intron-loxP-HPHMX Tom70-GFP-TRP1/+ VPH1-mCherry-Kan/+ sip2-K12R,K16R,K17R KanMX6-Pgdp-SAK1/+ acc1-S1157A/+* |
| MU88 | **ALD6Δ**  MEP diploid  (Tom70-GFP Vph1-mCherry) | *ade2::hisG his3 leu2 met15D::ADE2/+ lys2/+ ura3D0 trp1D63 hoD::SCW11pr-Cre-EBD78-NatMX loxP-UBC9-loxP-LEU2 loxP-CDC20-Intron-loxP-HPHMX Tom70-GFP-TRP1/+ VPH1-mCherry-Kan/+ ALD6::HIS* |
| MU1 | **WT-VPH1**  MEP diploid  (Tom70-GFP Vph1-mCherry) | *ade2::hisG his3 leu2 met15D::ADE2/+ lys2/+ ura3D0 trp1D63 hoD::SCW11pr-Cre-EBD78-NatMX loxP-UBC9-loxP-LEU2 loxP-CDC20-Intron-loxP-HPHMX Tom70-GFP-TRP1/+ VPH1-mCherry-Kan/+* |
