# Supplementary material for: Decoupling AMPK from fatty acid synthesis allows maintenance of fitness late in life
Source: eLife. 2026 Jul 31;15:RP111611. doi: 10.7554/eLife.111611 (PMC13427345; doi:10.7554/eLife.111611)
Supplement: Peer review history

# Editor report: eLife Assessment

**DOI:** 10.7554/eLife.111611.3.sa0

**Authors:** Weiwei Dang

**Affiliations:** Baylor College of Medicine United States

**Keywords:** Convincing, Important

This study addresses an **important** question in aging biology by combining metabolomics, transcriptomics, molecular genetics, and functional analyses to examine how cytosolic acetyl-CoA metabolism influences late-life fitness in replicatively aging yeast. The evidence supporting the roles of AMPK activation, mitochondrial acetyl-CoA utilization, and fatty acid synthesis in preserving fitness during aging is **convincing** overall, and the engineered A2A strain provides an elegant demonstration that coordinated modulation of distinct acetyl-CoA metabolic branches can increase the proportion of aged cells with a low-senescence phenotype. The study provides significant insight into mechanisms that allow aging cells to maintain fitness without extending replicative lifespan.

# Referee report: Reviewer #1 (Public review)

**DOI:** 10.7554/eLife.111611.3.sa1

This rigorous and creative study uses an elegant combination of metabolomics, transcriptomics, and budding yeast molecular genetics to discover that (i) activating AMPK to maintain mitochondrial respiration fuelled by cytosolic Acetyl CoA and (ii) increasing fatty acid synthesis independent of respiration drive independent pathways that increase the fitness of replicatively-aged budding yeast cells, albeit without increasing their lifespan. The reviewers have achieved their aims and the results support their conclusions. This work provides important insight into molecular mechanisms that allow aging without loss of fitness and will be of interest to scientists in the field of aging and metabolism.

# Referee report: Reviewer #2 (Public review)

**DOI:** 10.7554/eLife.111611.3.sa2

Summary:

In this study, the authors investigate how cytosolic acetyl-CoA metabolism influences replicative aging in budding yeast. They propose that acetyl-CoA regulates aging through three major pathways: (1) mitochondrial transport to support mitochondrial function, (2) fatty acid synthesis, and (3) global protein acetylation. The data show that AMPK activation promotes mitochondrial import of acetyl-CoA and partially mitigates mitochondrial decline in a subset of aging cells. Furthermore, the engineered A2A strain, which enhances mitochondrial acetyl-CoA utilization while relieving inhibition of fatty acid synthesis, increases the proportion of cells exhibiting a "low senescence" phenotype.

Overall, this is a thoughtful and potentially impactful study that advances our understanding of metabolic control of aging. Addressing the points below, particularly by refining interpretations and, where feasible, incorporating additional analyses, will further strengthen the manuscript and its conclusions.

Strengths:

The study has several notable strengths. It addresses an important question by shifting the focus from lifespan to preservation of late-life fitness, which is highly relevant to aging biology. The work integrates metabolic, genetic, and functional analyses to link cytosolic acetyl-CoA flux with distinct aging outcomes, and the engineering of the A2A strain provides a clear and elegant demonstration of how coordinated pathway modulation can improve cellular fitness.

Comments on revised version.

I am fine with the revisions.

# Author comment: Author response

**DOI:** 10.7554/eLife.111611.3.sa3

**Authors:** Hanane Hadj-Moussa, Megan Ulusan, Dorottya Horkai, Mohammed Kamran Afzal Mirza, Jonathan Houseley

**Affiliations:** The Babraham Institute Cambridge United Kingdom; The Babraham Institute Cambridge United Kingdom; The Maxwell Centre Cambridge United Kingdom; The Babraham Institute Cambridge United Kingdom; The Babraham Institute Cambridge United Kingdom

The following is the authors’ response to the original reviews.

**Public Reviews:**

**Reviewer #1 (Public review):**

This rigorous and creative study uses an elegant combination of metabolomics, transcriptomics, and budding yeast molecular genetics to discover that (i) activating AMPK to maintain mitochondrial respiration fueled by cytosolic Acetyl CoA and (ii) increasing fatty acid synthesis independent of respiration drive independent pathways that increase the fitness of replicatively-aged budding yeast cells, albeit without increasing their lifespan. This work will be of interest to scientists in the field of aging and metabolism. Some clarifications in the text would address the following concerns, which would increase the impact of the study:

(1) What does activation of AMPK (via PGDP-Sak1 expression) do to the replicative lifespan? How many bud scars, in general, do the subpopulations that are older - yet have less Tom70 (increased mitochondrial fitness) - have, after the 48 hrs timepoint that they are examining? How many divisions occurred in this 48hr time period - i.e. is it long enough to have all cells reach the end of their replicative lifespan? This information is important to rule out that a subset of the mutant cells just divided faster and hence had more divisions within 48 hrs (growing faster and living longer are different things). Having identical growth curves doesn't indicate per se that they all divide at the same rate, as there may be a subpopulation that divides faster and a subpopulation that doesn't grow so well.

Increasing AMPK activity increases replicative lifespan [PMID: 25869125], but given our finding that AMPK activation splits the population, such replicative lifespan assays are hard to interpret. Bud scar counts have a similar issue. Hence we restricted the lifespan and bud scar analyses to wt and A2A which are more homogenous (Figures S2 B and E). A2A cells at 48 h have ~25% more bud scars than wt cells. Yes, by 48 h most of the cells have lost viability (Figure 2E). The reviewer is correct that you can't properly compare the lifespan curves if the cells divide at different rates, hence our follow-up test of wt at 48 h vs A2A at 40 h viability after we had confirmed that these time points captured cells at equivalent replicative ages (Figure 2D, E). This shows that viability of A2A is slightly lower than wt at matched age, indicating a slightly shorter lifespan.

(2) A2A cells do not have an extended replicative lifespan (RLS) but show an increase in the "low senescence" population (Figure 2). If the cells are not becoming senescent, why don't they have longer RLS? Not having a longer lifespan seems inconsistent with the statement that "bud scar counting confirmed that A2A cells reach a higher age than wild type", which comes back to how many times the cells can divide in the 48hr timepoint studied and their rate of cell division? Also, the lifespan curve shown is plotted against time, not cell division number, which does not take into account different division times of cells within the population (described above). It would be much more useful to show standard lifespan curves showing cell division numbers per lifespan per cell.

Our observation that cells can reach the end of life without senescing is consistent with other studies that have studied the life course of individual cells by microscopy [PMID: 31291577, 32675375]. These studies always highlight some proportion of the cells that reach the end of life with no or minimal senescence, though this fraction varies with the experimental system. The question of why cells lose viability without senescing is a complete unknown in the field, and reflects a wider lack of consensus as to why yeast lose viability with replicative age.

In liquid culture we can only assess viability over time, not cell division number, which we agree is not optimal and we are wary about making strong statements on lifespan for exactly the reasons the reviewer notes. Unfortunately, it is clear from the comparison of liquid and solid media lifespans performed by the Gottschling lab [PMID: 19652178] that culture system has a huge effect on lifespan, with cells in classical plate-based microdissection assays living far longer than the same strains do in liquid. This means that lifespans determined by microdissection-based assays are of questionable relevance to ageing studies performed in liquid culture. Senescence cannot be assayed on plates, while microfluidic systems lack the throughput necessary and preclude key techniques like RNA-seq, so liquid culture assays were the only option for this work. We agree that this leaves an unsatisfactory approximation for lifespan measurements, but we consider it critical that everything is measured in the same system. We therefore restricted our conclusion on lifespan to simply say that lifespan of A2A cells is not extended which our data in Figures 2D, E, S2B does support (see also answer to Q1), and therefore with the majority of A2A cells showing low senescence marks and high fitness at 48 h we can conclude that lifespan and fitness loss must be separable.

We have added a note of these limitations of lifespan measurements in the materials and methods section of the manuscript.

(3) Increased "fitness" of the old cells is implied from the increased size of the colonies that the old cells can make. However, this is a measure of the fitness of the daughters per se, not the old mother cells. Are the old mothers just passing on healthier mitochondria and more lipids to the daughters, such that they can divide more times? If the aged cells have an "increased fitness", why don't they divide more times themselves (i.e. live longer?).

Yes, colony growth speed is defined by daughter cell replication, but as long as the daughters and subsequent generations divide at the same rate irrespective of whether they come from a young or old mothers then the size of the colony after 24 hours varies based on the time it took the initial mother to produce a daughter. This is what the assay really measures. We note that aged wildtype mothers often do not divide at all in the first 24 hours after being put on an agar plate (hence the tiny reported colony size), even though they do eventually produce a daughter which then forms a colony, whereas A2A cells tend to produce the first daughter rapidly whether young or old. It is known that daughters of aged wildtype mothers also divide slower, as to some extent do grand-daughters (PMID: 2644196), which will also contribute to differences in colony size, and this may well result from a lipid and/or mitochondrial contribution, but the primary driver of colony size in 24 hours is the time the mother took to initially divide. We have added this detail to the materials and methods section of the manuscript.

As noted above, the mechanistic basis of lifespan is unknown, but although senescence can shorten lifespan, our work and that of others shows that lifespan is still limited in the absence of senescence.

(4) The statement is made that "these experiments define two classes of aging cells with distinct metabolic needs, coherent with the model of two aging trajectories previously proposed (referencing Nan Hao's work)". However, the big difference here is that in Nan Hao's work, their two aging trajectories influenced the length of lifespan, but that does not appear to be the case here. That distinction should be made clear. Perhaps the authors could also speculate as to why the A2A yeast stops dividing after presumably the same number of cell divisions, even though they have an activated AMPK and activated fatty acid synthesis pathway.

Yes, this is a good point and we have added this distinction to the Discussion:

“Here we have characterised two classes of ageing cells seemingly differentiated by high and low availability of cytosolic Acetyl-CoA, consistent with a previous demonstration that ageing follows two trajectories in yeast though it should be noted that in this previous report, the two trajectories also differed in replicative lifespan (6).”

We would love to speculate on why the A2A cells don't have an extended lifespan, but at this point we don't have a strong hypothesis. We have come up with many theories for this, but none that we haven’t managed to disprove experimentally. One thing worth considering is that many cells which lose replicative viability in liquid culture and probably in plate assays remain intact – for example, DNA and RNA integrity is not compromised over 24- 48 h – so those cells are probably not dead per se. But we also detect apoptosis-sized DNA fragments, which must come from dead cells, so there is clearly not a single mechanism defining the end of replicative lifespan.

(5) I am a bit confused by the use of the word "senescence" by this lab here and in their previous growth on galactose studies. If yeast don't senesce, which is usually defined as an irreversible arrest of the cell cycle where cells stop dividing, shouldn't the yeast that do not senesce still be dividing and hence have a longer lifespan? Should a different term be used rather than senescence? Such as "fitness late in life". The authors giving their definition of senescence may help reduce this apparent contradiction.

We completely agree, this is confusing and noted this distinction in the Introduction. Use of the term senescence to mean a loss of fitness late in life in yeast stems from the classical definition of senescence as applied to whole organisms. However, the term senescence as applied to cells has a more specific meaning in terms of the cell cycle as the reviewer notes. As an individual *S. cerevisiae* is both a cell and an organism, the terminology clashes. However, the marker we largely employ (Tom70-GFP) which in our hands is a very good proxy for fitness was originally defined as marking the senescence entry point (SEP), so overall we feel we can't avoid the term.

**Reviewer #2 (Public review):**

Summary:

In this study, the authors investigate how cytosolic acetyl-CoA metabolism influences replicative aging in budding yeast. They propose that acetyl-CoA regulates aging through three major pathways: (1) mitochondrial transport to support mitochondrial function, (2) fatty acid synthesis, and (3) global protein acetylation. The data show that AMPK activation promotes mitochondrial import of acetyl-CoA and partially mitigates mitochondrial decline in a subset of aging cells.

Furthermore, the engineered A2A strain, which enhances mitochondrial acetyl-CoA utilization while relieving inhibition of fatty acid synthesis, increases the proportion of cells exhibiting a "low senescence" phenotype.

Overall, this is a thoughtful and potentially impactful study that advances our understanding of metab to olic control of aging. Addressing the points below, particularly by refining interpretations and, where feasible, incorporating additional analyses, will further strengthen the manuscript and its conclusions.

Strengths:

The study has several notable strengths. It addresses an important question by shifting the focus from lifespan to preservation of late-life fitness, which is highly relevant to aging biology. The work integrates metabolic, genetic, and functional analyses to link cytosolic acetyl-CoA flux with distinct aging outcomes, and the engineering of the A2A strain provides a clear and elegant demonstration of how coordinated pathway modulation can improve cellular fitness.

Weaknesses:

(1) While the manuscript focuses on mitochondrial transport and fatty acid synthesis, cytosolic acetyl-CoA is also a key regulator of histone acetylation and chromatin silencing. It would strengthen the study to consider whether acetyl-CoA depletion contributes to improved fitness through enhanced rDNA silencing. Given the well-established role of rDNA instability in yeast aging, additional experiments examining rDNA silencing and stability would be valuable. For example, monitoring rDNA copy number changes (not necessarily ERCs) under AMPK activation, oleic acid supplementation, and in the A2A strain, similar to approaches used in the authors' prior work, would help clarify whether chromatin regulation contributes to the observed phenotypes.

We have added data addressing these points to the manuscript and Supplemental Figures 2, 3 and 4, though the outcomes are complex. Histone acetylation changes chromatin accessibility and could therefore alter global gene expression; in accord with this, RNA-seq shows that *P_GPD_-SAK1* reduces known age-linked gene expression dysregulation. However, A2A does not further reduce the effect, meaning either that another driver exists in addition to cytosolic acetyl-CoA, or that age-linked gene expression dysregulation is unrelated to cytosolic acetyl-CoA. Oleic acid has little effect on age-linked gene expression dysregulation despite rescuing fitness. With regard to rDNA silencing, transcription of the rDNA intergenic spacer non-coding RNAs promotes ERC formation; we have added data showing that ERC accumulation is not reduced in A2A but slightly higher coherent with the higher replicative age of A2A at 48 h, which suggests silencing is not better in A2A. By RNA-seq, these intergenic spacer transcripts are massively upregulated with age, but this will be a consequence of the increased genomic copy number on ERCs; the upregulation is less in A2A than other conditions, but this arises because the log phase spacer transcript levels are higher and so does not reflect better rDNA silencing. We have previously assayed for heritable changes in rDNA copy number arising during ageing and found (to our surprise) absolutely nothing, so we don't expect any changes under these conditions. The upregulation of transcripts from Sir2-repressed telomeric and MAT loci with age is decreased in *P_GPD_-SAK1* and A2A, but the effect size is not different from any other low-expressed genes so we do not think there is a particular effect at loci subject to chromatin silencing (see our previous study Zylstra et al PMID 37643194 for evidence that Sir2-mediated gene silencing is not affected by age). We have added our conclusions from these experiments to the Discussion.

(2) The current data do not fully distinguish whether AMPK activation and oleic acid supplementation act on distinct subpopulations of aging cells. An alternative explanation is that oleic acid supplementation enhances mitochondrial function and acts additively with AMPK activation, thereby increasing the fraction of cells in the "low senescence" state. Since this distinction is not central to the main conclusions, I suggest softening the language around subpopulation specificity. Emphasizing instead that the A2A strain coordinately modulates multiple branches of acetyl-CoA metabolism to improve late-life fitness would maintain the strength of the central message without over interpretation.

We respectfully disagree with the reviewer on this point. We show that *P_GPD_-SAK1* rescues senescence in ~half the population by a Cat2/Mls1 dependent mechanism (Figure 1F). We then show that in A2A, which rescues most cells, deletion of *CAT2/MLS1* restores senescence in ~half the cells (Figure 3F/G). This cannot be explained by an additive mechanism as this would either result in all cells being partially rescued in the *P_GPD_-SAK1* and in the A2A *cat2*Δ *mls1*Δ mutants, which is definitely not the case either by Tom70-GFP or fitness. Instead the population splits into high/low senescence and fit/unfit cells in the different assays.

On the specific point of whether lipid synthesis additively increases mitochondrial function, we have added oxygen consumption rate data showing that A2A cells respire more than *P_GPD_-SAK1* at 48h but only by a relatively small amount (Figure S3D), so there is indeed an additive improvement in mitochondrial function, but too little to explain the difference in population fitness in our opinion.

We realise that the reviewer is asking more specifically about oleic acid, but again in the flow data, Figure 4C, what changes with oleic acid or *P_GPD_-SAK1* is the proportion of cells in the low Tom70 / high WGA sector. Under an additive effect model, oleic acid or *P_GPD_-SAK1* individually would partially reduce Tom70 and partially increase WGA, but the population in the low Tom70 / high WGA sector has the same average Tom70/WGA values in oleic acid, *P_GPD_-SAK1* or *P_GPD_-SAK1*+oleic acid. It is the proportion of cells in this population that changes. Furthermore, under an additive model, wildtype cells aged with oleic acid would not have highest fitness than *P_GPD_-SAK1* or A2A (Figure 4D) as these individual cells would lack the mitochondrial upregulation from *P_GPD_-SAK1*.

(3) The manuscript proposes that lipid starvation and excess acetyl-CoA are major drivers of senescence in distinct subpopulations of wild-type aging cells. This conclusion is not yet fully supported by the presented data. Direct measurements of age-dependent divergence in acetyl-CoA and fatty acid levels at the single-cell level would be needed to substantiate this model. Based on the current evidence, a more conservative interpretation would be that aging cells exhibit differential sensitivity to perturbations in acetyl-CoA and lipid metabolism. Accordingly, I recommend revising the statement in the Abstract ("We further implicate lipid starvation and excess acetyl coenzyme A availability as major drivers of senescence...") and the corresponding discussion text to better align with the data.

We agree and have adjusted the abstract to make it clearer that the lipid starvation / excess acetyl-coA interpretation is a model.

“Our findings support a model in which lipid starvation and excess acetyl-coenzyme A availability are major drivers of senescence in replicatively aged wild-type yeast.”

**Reviewer #3 (Public review):**

Summary:

These findings suggest that PGPD-SAK1 yeast show a subpopulation with lowered TOM70-GFP expression in high bud scar staining aged cells. Deletion of CAT2 or MLS1 reduces this effect. A PGPD-SAK1 acc1S1157A double mutant (called "A2A" here) shows an even larger effect of lowered tom70 expression in high bud scar staining aged cells. Utilization of various additional mutants involved in acetyl-CoA transport, carnitine shuttle, respiration, etc., leads the authors to conclude that these shifts in TOM70-GFP in aged cells are linked to the AMPK-fatty acid metabolic regulatory system.

Strengths:

These extensive and clearly described experiments reveal interesting changes in TOM70-GFP intensity in subsets of aged yeast in several mutants eventually identified as linked to the AMPK-fatty acid metabolic regulatory system.

Weaknesses:

(1) 3 biological replicates for mRNASeq is low.

Thank you for pointing this out. We performed another replicate after posting the initial preprint to confirm the finding but didn’t update the figure in the eLife-reviewed version. We have added this to the scatter plots and analysis in Figure 1, there are minor changes but the set of genes we followed up are still highly significant. For ageing experiments, we sequence to n=3 as a first pass which is sufficient to detect widespread age-linked gene expression effects, and add more replicates if required to solidify findings for specific sets of genes. Hence, the additional RNAseq experiments we have added to the manuscript to Address Reviewer 2’s comments on widespread gene expression effects are also n=3-4.

(2) While "Traditional conceptions of ageing implicate a progressive accumulation of damage leading to systemic degradation in performance until death, with evolutionary pressures acting to maximise early life fitness and fecundity at the expense of ageing health." is tangential perhaps to the data and conclusions of the study, both claims of this sentence are at best controversial, and the manuscript is no weaker for their omission.

We would prefer not to remove this sentence, which we see as important to a major message of the manuscript: that ageing does not have to involve a loss of fitness before death. Outside the ageing biology field, ageing is often described as the progressive wearing out of components leading to decline and death (‘like an old car’ is a common analogy); in the ageing field this is certainly controversial, but outside the field it remains the normal understanding. This is what we mean by traditional conceptions, and it is important to consider the contradiction between this widely held viewpoint and our findings (and of course those of many others in the ageing field).

The second part of the sentence about evolutionary pressures alludes to antagonistic pleiotropy, which we have now made explicit. Antagonistic pleiotropies as a driving mechanism for ageing, while not universally accepted, are as far as we can tell the most widely accepted type of theory in the ageing field. Our interpretation that yeast are bet-hedging as a population growth strategy and this drives ageing in the long term is a classic antagonistic pleiotropy and we need to raise this concept in the introduction.

(3) The statement that "Here, we determine the basis of senescence and fitness loss in replicatively ageing yeast" is a bit strong as a summary of the present careful work presented here. If the authors had created yeast mutants that retained fitness indefinitely, this would be a more appropriate strength of claim to summarize the work.

We agree and have moderated this sentence:

“Here, we show that senescence and fitness loss in replicatively ageing yeast can be almost completely avoided without extension of lifespan by rewiring the conserved AMPK-fatty acid metabolic regulatory system.”

**Recommendations for the authors:**

**Reviewer #1 (Recommendations for the authors):**

The labelling of Figure 3G horizontal axis needs to be realigned with the data.

Fixed – thank you.

**Reviewer #2 (Recommendations for the authors):**

(1) In Figure 3G, the x-axis labels appear misaligned and should be corrected for clarity.

Fixed – thank you.

(2) Figures S3B and S3C appear to be mislabeled and should be revised.

Fixed – thank you.

(3) On page 6 (3rd paragraph), the statement that the beneficial impact arises from acetyl-CoA removal "rather than a benefit of respiration" may be overstated. The data support a role for acetyl-CoA removal but do not fully exclude a contribution from respiration. A more balanced phrasing would improve accuracy.

We have revised this sentence and also added data:

“Working in sip2Δ to avoid an increase in AMPK activity due to reduced Acetyl-CoA availability, we observed that ald6Δ increased the low senescence population through decreasing Tom70-GFP (S3C), and therefore the beneficial impact of PGPD-SAK1 on this pathway arises primarily through Acetyl-CoA removal. It is possible that respiration is adding to this benefit, and we detect a significant increase in Oxygen Consumption Rate in aged PGPD-SAK1 cells, but the further increase in A2A is smaller and we consider that this cannot fully explain the effect of acc1S1157A.”

**Reviewer #3 (Recommendations for the authors):**

This manuscript is clearly written, and the data are clearly presented. While 3 biological replicates is inadvisably low for mRNASeq, the subsequent experiments motivated by the genes identified there nevertheless stand on their own as presented.

Thank you.
